# Is submuscular drainage mandatory for posterior spinal fusion in adolescent idiopathic scoliosis? A retrospective clinical study

**DOI:** 10.3389/fsurg.2026.1816291

**Published:** 2026-04-29

**Authors:** Duan Wenbo, Fang Guofang, Wu Jiachang, Sang Hongxun, Cao Lei

**Affiliations:** 1 Department of Orthopaedics, China-Japan Union Hospital of Jilin University, Changchun, Jilin Province, China; 2 Department of Orthopaedics, Shenzhen Hospital of Southern Medical University, Shenzhen, Guangdong Province, China

**Keywords:** adolescent idiopathic scoliosis, intracavitary hematoma, lumbar mobility, posterior spinal fusion, submuscular drainage

## Abstract

**Objective:**

This single-center retrospective analysis was designed to evaluate the outcome of closed-suction wound drainage following posterior spinal fusion with internal instrumentation for mild to moderate adolescent idiopathic scoliosis (AIS).

**Methods:**

Eighty-six AIS patients undergoing posterior spinal fusion were divided into two cohorts: submuscular closed-suction wound drainage (*n* = 35) and simple compressed dressing clothes without wound drainage (*n* = 51). These two cohorts were thoroughly compared in terms of demographic distribution and perioperative blood loss, including hemoglobin and hematocrit levels and blood transfusion volumes. Additionally, the incidence of wound-related problems (pyrexia and wound complications), duration of hospital stay, and lumbar function evaluation (lumbar mobility and SRS-22 questionnaire scores) were annually assessed during at least 5-year follow-up.

**Results:**

The drainage group had significantly lower hemoglobin (93.73 g/L vs. 99.95 g/L, *P* = 0.01) and hematocrit levels (27.75% vs. 29.94%, *P* < 0.01) on the third postoperative day, as well as a significantly higher postoperative blood transfusion volume (40.0 mL vs. 23.5 mL, *P* = 0.011) compared to the non-drainage group. Furthermore, the duration of hospital stay was significantly longer in the drainage group than in the non-drainage group (10.9 d vs. 8.0 d, *P* < 0.01). In contrast, the two groups were statistically similar regarding duration of fever (0.9 d vs. 1.2 d, *P* = 0.268), incidence of wound problems, latest lumbar mobility (42.79° vs. 44.97°, *P* = 0.586), and scores of function/activity domain (16.74 vs. 16.08, *P* = 0.285) and pain domain (22.18 vs. 21.48, *P* = 0.374) in the SRS-22 questionnaire.

**Conclusions:**

Routine closed-suction drainage significantly increased blood loss and hospital stay without obviously improving wound healing or functional outcomes. Utilizing simple compressed dressings without drainage was a clinically superior and resource-efficient alternative for posterior AIS fusion, particularly in uncomplicated primary surgeries for mild to moderate deformities.

## 1 Introduction

Closed-suction wound drainage has been extensively applied in spine surgeries. Theoretically, wound drainage could effectively facilitate wound healing by providing a ready egress away from the surgical site, minimize the potential compression of exposed nerve structures, and reduce the risk of surgical site infection (1, 2). However, the aforementioned advantages have also been questioned by a number of researchers (3, 4). Some researchers argued that closed-suction drainage had limited benefits in minimizing hematoma as well as in preventing further compression of the spinal cord (4). In addition, the potential disadvantages of drainage, such as increased perioperative blood loss, canal-related contamination, prolonged hospital stay and increased nursing workload, have been frequently reported (5, 6). Hitherto, there has been no firmly established consensus on indications for drainage placement, drainage type and drainage depth and duration. Many spine surgeons even decided drainage placement and patterns based on personal preferences arbitrarily (5, 7).

Adolescent idiopathic scoliosis (AIS) is a complex 3-dimensional spine deformity involving coronal, sagittal and axial malalignment (8). Compared with regular spine surgery, the posterior spinal fusion with instrumentation in AIS is characterized by larger surgical sites, more blood loss and more complex postoperative complications. Moreover, the extensive dissection of paravertebral muscle and decortication of vertebral laminae make hemostasis more challenging (9). Particularly, effective postoperative drainage is capable of minimizing the hematoma formation and organization that could lead to reduced lumbar mobility (10). Nevertheless, the risk of drainage-related infection and potential blood loss cannot be neglected (11). To date, there are few high-quality studies discussing the influence of wound drainage on back pain and lumbar mobility in AIS patients. In this retrospective clinical study, we carried out a thorough comparison of perioperative drainage-related indices and long-term lumbar mobility.

## 2 Materials and methods

This retrospective clinical study was approved by the Institutional Review Board of the hospital where a number of sophisticated orthopedic surgeons were employed. Two groups of AIS patients undergoing posterior fusion and internal fixation were retrospectively and consecutively enrolled. Group A (drainage group) included 35 patients who received postoperative submuscular drainage accompanied with thick compressed dressings, and Group B (non-drainage group) consisted of 51 patients who received compressed dressings purely. All surgical procedures were performed by a single team of skilled orthopedic surgeons from June 2015 to September 2020.

### 2.1 Patients

The inclusion criteria for eligible patients were as follows: (1) aged 10–18 years and diagnosed with AIS according to the Lenke classification system (12), (2) presenting a major thoracic or lumbar/thoracolumbar curve less than 80°, (3) treated with pedicle screw and rod instrumentation via posterior approach, (4) followed up for at least 5 years at intervals of 6 months. Within the drainage group, there were 4 females and 31 males with an average age of 14.8 years and a mean follow-up period of 62.0 months (from 58 to 67 months). As for the non-drainage group, there were 43 females and 8 males, with an average age of 15.9 years and a mean follow-up period of 65.4 months (from 60 to 71 months).

### 2.2 Strategy of fusion extent

All patients were treated with pedicle screws and rod instrumentations through posterior approach. The upper end vertebra (UEV) was selected as upper instrumented vertebra (UIV). The lower instrumented vertebra (LIV) was the last substantially touching vertebra (LSTV) as was described by Zhu's group (13).

### 2.3 Surgical procedures

Exposure: We placed the patient in a prone position and made a midline incision on the back. The epidermis, subcutaneous tissue and paravertebral muscles were cut to expose the vertebrae at the planned fusion levels.

Pedicle screws placement: Pedicle screws were placed at each level on the convex side and at every 1–3 levels on the concave side using a freehand technique. Then the inferior articular process was removed for stress release and better fusion.

Rod placement and locking: We installed a prebent correcting rod on the convex side of the major curve, secured the nuts onto the screws, and rotated the rod to rebuild the normal sagittal alignment. Appropriate distraction and compression force were applied on the major curve and then screws were locked after each distraction or compression maneuver. Subsequently, we embedded a prebent maintenance rod on the concave side after achieving a satisfactory outcome. The rod on the concave side was tightened after appropriate maneuvers in a similar way. Necessary adjustments were performed via *in situ* benders according to intraoperative shoulder balance assessment and the C7-central sacral vertical line (C7-CSVL).

After finishing the correction, one or two transverse connectors were installed. The laminae of instrumented levels were decorticated and autograft and allograft bones were added to promote bone fusion.

Intraoperative motor evoked potential (MEP) and somatosensory evoked potential (SSEP) were used in all patients to monitor spinal cord function.

### 2.4 Postoperative management

**Figure 1:**
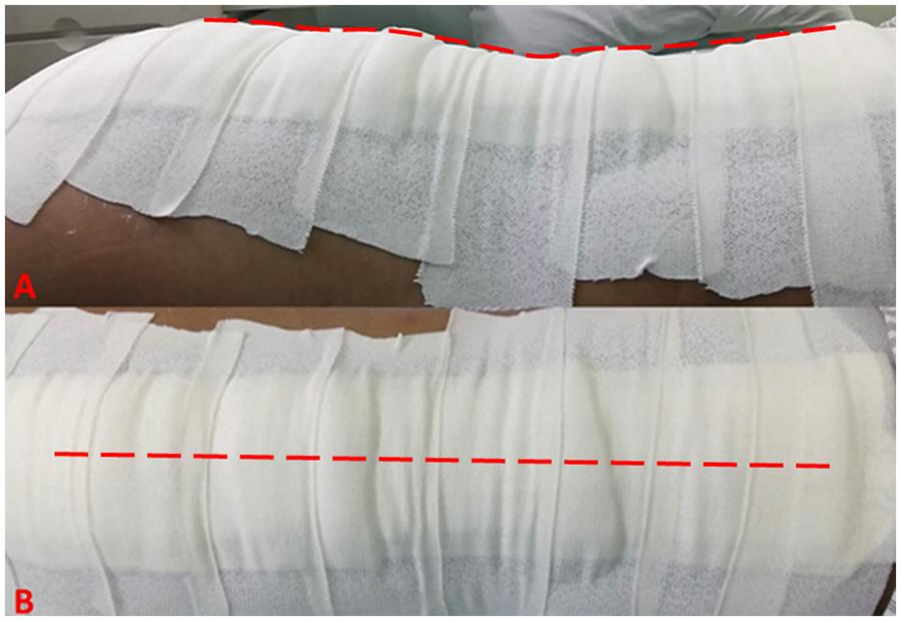
**(A)** The surgical incision was covered with the typical compressed dressing clothes, the upper border of which was approximately paralleled with the horizontal line. **(B)** The thick dressing clothes were stuck to the skin by elastic adhesive tapes in a symmetric pattern.

All patients were equipped with thick compressed dressing clothes on surgical sites (Figure 1) and were encouraged to perform bedside rehabilitation exercise with the protection of hard bracing. The drainage tube was removed when daily drainage volume was less than 50 mL/d. Red blood cell (RBC) transfusion therapy was applied when Hb level was less than 70 g/L or typical anemia-related dizziness, dyspnea or tachycardia were observed (14). All patients were instructed to take off the bracings after 3 months and started physical exercise training under the guidance of a single group of physical therapists.

**Figure 2:**
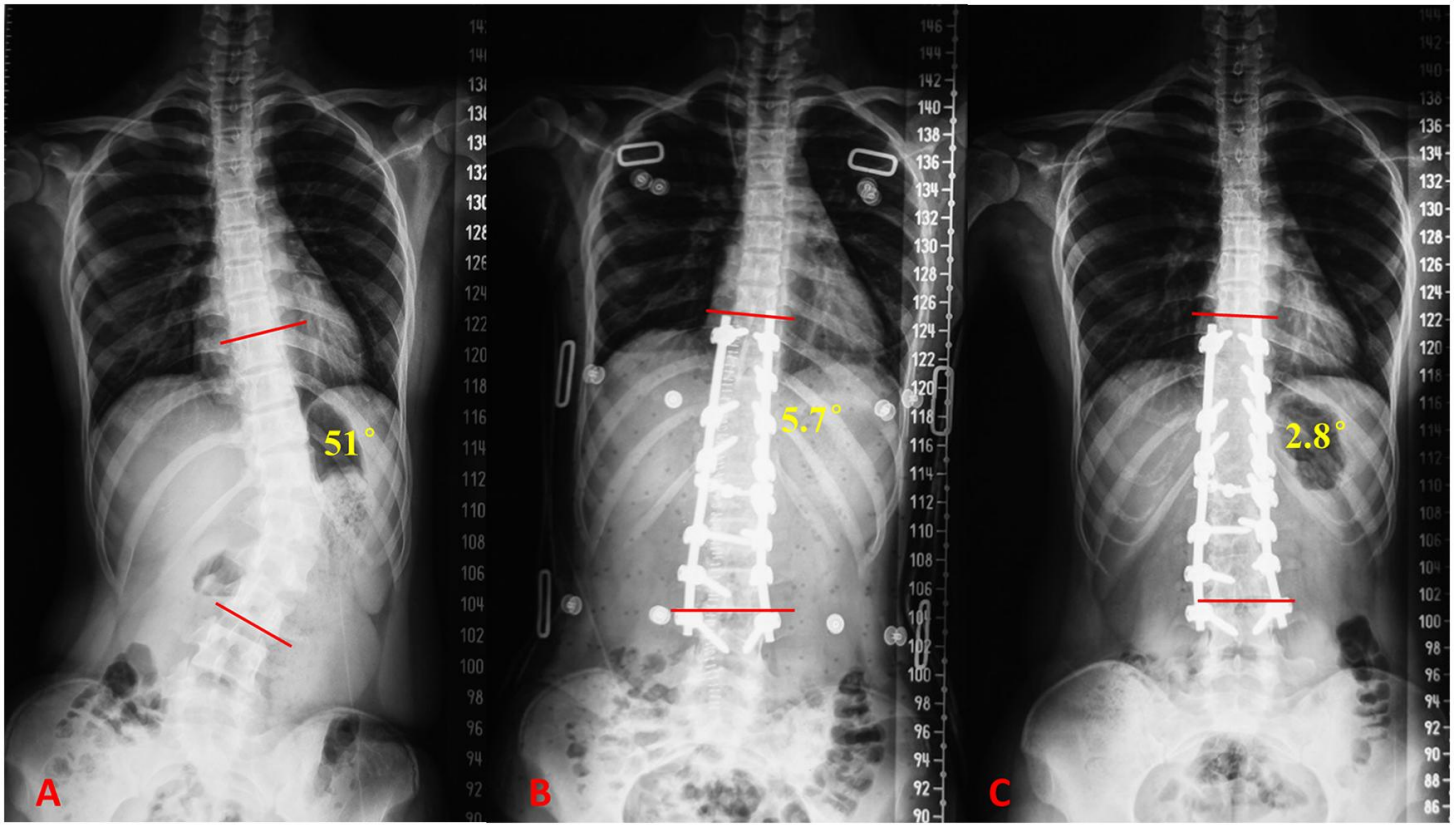
**(A)** A 14-year-old female patient was diagnosed as Lenke 5C AIS, with a major lumbar curve of 51°. **(B)** The major cobb angle was corrected to 5.7° immediately after surgery and no submuscular drainage was placed. **(C)** The major cobb angle was spontaneously improved to 2.8°at the latest follow-up. No myositis ossificans was observed on x-ray films.

All patients were followed up for at least 5 years and were annually guided to complete the SRS-22 questionnaires independently according to their subjective feelings. We also measured lumbar mobility at each follow-up repetitively with the help of a digital inclinometer following the method reported by Sanchez's (15). A typical case was shown in Figure 2.

### 2.5 Statistical analysis

The perioperative parameters included: demographic data (including age, gender, height, weight, body mass index), preoperative data (pre-op Cobb angle, Lenke classification, pre-op Hb and Hemocrit level), intraoperative data (operation time, fusion segments, estimated blood loss, intra-op blood transfusion volume), postoperative data (post-op blood transfusion volume, post-op Hb and Hemocrit level, duration of pyrexia, peak body temperature, surgery-related complication, length of hospital stay). The long-term parameters included: follow-up times, function/activity domain and pain domain scores in SRS-22 questionnaires.

Data were analyzed using Statistical Package for the Social Sciences software (IBM SPSS Statistics, RRID: SCR_016479). Since this study was a single-center retrospective cohort study with minimally 5-year follow-up, it was difficult to estimate the sample size in advance. Therefore, we performed a *post-hoc* power analysis for the key outcomes (e.g., intraoperative data and postoperative clinical and hematologic data, etc.) to calculate the study's power based on the final sample size. The normal distribution of continuous variables was assessed using the Kolmogorov–Smirnov test, while homogeneity of variance was evaluated using Levene's test. Continuous variables with normal distribution and homogenous variance were analyzed using bilateral independent *t*-tests if the corresponding variables were measured simultaneously. Otherwise, Wilcoxon rank-sum tests were applied. For SRS-22 questionnaire scores, we utilized MANOVA analysis given that each domain was influenced by multiple factors (e.g., ages, subjective feelings, missing values, etc.). Moreover, Fisher exact test and Pearson Chi-square test were used for categorical variables.

## 3 Results

### 3.1 Preoperative data

The preoperative data are shown in Table 1. No significant difference was found between the two groups in age, gender, height, weight, body mass index (BMI), preoperative Cobb angle and Lenke curve type.

**Table 1:** Preoperative characteristics of two groups (mean ± SD).

Demographic data	Group A	Group B	*P* value
	(*n* = 35)	(*n* = 51)	
Age (y)	14.8 ± 3.2	15.9 ± 2.6	0.081^a^
Male/Female	4/31	8/43	0.256^a^
Height (cm)	155.3 ± 8.7	156.8 ± 10.5	0.533^a^
Weight (kg)	43.2 ± 7.2	44.8 ± 7.3	0.319^a^
BMI (kg/m^2^)	17.8 ± 2.2	18.5 ± 5.0	0.459^a^
Preoperative main curve Cobb angle (°)	54.6 ± 14.3	50.9 ± 12.2	0.055^a^
Lenke curve type, *n* (%)			0.455^a^
Type I	17 (48.6)	26 (51.0)	
Type II	1 (2.8)	5 (9.8)	
Type III	1 (2.8)	2 (3.9)	
Type IV	2 (5.7)	2 (3.9)	
Type V	8 (22.9)	11 (21.6)	
Type VI	6 (17.2)	5 (9.8)	

Group A, compressed dressing clothes with submuscular closed suction drainage; Group B, compressed dressing clothes with no drainage.

a Compared with group A, *P* > 0.05.

### 3.2 Intraoperative data

We calculated the effect size of each representative indicator of intraoperative and postoperative data using Cohen's d $(d=\frac{Mean1-Mean2}{Sp}$, $Sp=\sqrt{\frac{(n1-1)S1^{2}-(n2-1)S2^{2}}{n1+n2-2}})$. Based on the effect size, we further calculated a statistical power of at least 0.802 to detect the observed difference at *α* = 0.05. The normal distribution and homogeneity of the key perioperative data were confirmed by the Kolmogorov–Smirnov test and Levene's test, respectively. Generally, there were no significant differences between the two groups regarding operation time, estimated blood loss (EBL), autologous blood transfusion volume, allogenic blood transfusion volume, number of fusion segments and pedicle screws (Table 2). No osteotomy was performed and no intragenic complications such as cerebrospinal fluid leakage or pleural rupture occurred during surgeries. No abnormal MEP or SSEP signal change was observed during pedicle screw insertion and correction maneuver.

**Table 2:** Intraoperative data of the patients with different fusion extent (based on lumbar fusion) (mean ± SD).

Intraoperative data	LIV ≥ L1		*P* value	(LIV < L1)		*P* value
	Drainage	Non-drainage		Drainage	Non-drainage	
	(*n* = 8)	(*n* = 9)		(*n* = 27)	(*n* = 42)	
Operation time (min)	248.1 ± 54.7	235.7 ± 45.1	0.614^a^	266.8 ± 46.6	253.2 ± 41.9	0.205^a^
EBL (mL)	612.5 ± 231.7	640.2 ± 203.6	0.347^a^	676.3 ± 356.9	664.9 ± 293.6	0.885^a^
Autologous blood transfusion (mL)	475.0 ± 204.8	448.9 ± 175.3	0.459^a^	535.0 ± 244.8	488.9 ± 205.3	0.300^a^
Allogenic blood transfusion (mL)	40.0 ± 106.3	27.4 ± 80.2	0.534^a^	38.7 ± 108.6	26.3 ± 68.5	0.566^a^
Allogenic blood transfusion cases, *n* (%)	1 (12.5%)	1 (11.1%)	0.692^a^	4 (14.8%)	5 (11.9%)	0.492^a^
NO. of fused segments	11.9 ± 0.8	11.8 ± 1.8	0.894^a^	12.8 ± 2.4	12.4 ± 2.8	0.527^a^
NO. of pedicle screws	15.00 ± 2.56	15.65 ± 2.30	0.644^a^	15.55 ± 2.85	15.68 ± 3.05	0.850^a^
NO. of reserved lumbar segments	5.00 ± 0.76	4.74 ± 0.53	0.296^a^	2.29 ± 0.69	2.32 ± 0.66	0.877^a^

a Compared with drainage group, *P* > 0.05.

To investigate the underlying mechanism of wound drainage in different surgical sites, we further divided the patients into two subgroups according to whether the lumbar segments were extensively fused (LIV < L1) or not (LIV ≥ L1). When evaluating the aforementioned intraoperative data in the patients whose LIVs were above L1 (*n* = 17), the difference between the drainage (*n* = 8) and non-drainage (*n* = 9) cohorts was insignificant (Table 2). A similar outcome was obtained in the patients whose LIVs were below L1 (*n* = 69) (Table 2).

### 3.3 Postoperative data

Table 3 presents the postoperative data of the two groups in general. Characterized by an obviously longer hospital stay (10.9 ± 3.6 d vs. 8.0 ± 2.6 d, *P* < 0.01), the Group A also received a greater postoperative blood transfusion volume (40.0 ± 24.7 mL vs. 23.5 ± 15.1 mL, *P* = 0.011) and exhibited a higher blood transfusion rate (7/35 vs. 5/51, *P* = 0.028). We also recorded and listed the hemoglobin and hemocrit levels as the two important indices that indirectly reflected postoperative blood loss (Table 4). The hemoglobin and hemocrit values in the drainage group were significantly lower compared with non-drainage group on the third postoperative day (93.73 ± 10.08 g/L vs. 99.95 ± 9.45 g/L, 27.75 ± 2.55% vs. 29.94 ± 3.15%, *P* < 0.01). Similar outcomes were observed in the patients whose lumbar segments were extensively fused (LIV < L1) (Table 5). The hospital stay duration and postoperative blood transfusion were significantly higher in the drainage cohort than in the non-drainage cohort (10.9 ± 3.8d vs. 7.7 ± 2.1d, 45.16 ± 88.84 mL vs. 21.05 ± 62.20 mL, *P* < 0.01). Meanwhile, the differences in the 3-day hemoglobin and hemocrit values were statistically significant (92.51 ± 7.64 g/L vs. 99.12 ± 9.81 g/L, 27.52 ± 1.93% vs. 29.79±3.35%, *P* < 0.01) (Table 4). Conversely, in the patients whose lumbar segments remained unfused (LIV ≥ L1), no significant difference existed between the drainage and non-drainage group with respect to postoperative indices (Table 4). Generally, no significant difference was found in the duration of postoperative pyrexia (axillary temperature ≥ 37.3 ℃) (1.1 ± 0.7d vs. 1.2 ± 0.6d, *P* = 0.268) and the peak temperature value within 72 h (37.5 ± 0.4 °C vs. 37.6 ± 0.4 °C, *P* = 0.523), indicating that the two groups presented an approximately similar degree of aseptic inflammatory reaction as well as febrile reaction (Table 3). The incidences of hematoma-related complications were similarly low in both groups (3 swellings in Group A, 2 swellings and 2 ecchymosis in Group B). All these cases received no targeted intervention and recovered spontaneously before discharge (Table 3).

**Table 3:** Postoperative clinical data of the patients of two groups (mean ± SD).

Postoperative data	Group A	Group B	*P* value
	(*n* = 35)	(*n* = 51)	
Length of stay (days)	10.9 ± 3.6	8.0 ± 2.6*	<0.01^b^
Duration of pyrexia (days)	1.1 ± 0.7	1.2 ± 0.6	0.268^a^
Peak temperature within 72 h (°C)	37.5 ± 0.4	37.6 ± 0.4	0.523^a^
Allogenic blood transfusion cases, *n* (%)	7 (20%)	5 (9.8%)*	0.028^b^
Blood transfusion volume (mL)	40.0 ± 24.7	23.5 ± 15.1*	0.011^b^
3rd-day Hemoglobin (g/dL)	93.73 ± 10.08	99.95 ± 9.45*	<0.01^b^
3rd-day Hematocrit (%)	27.75 ± 2.55	29.94 ± 3.15*	<0.01^b^
Incision-related complications, *n* (%)	3 (8.57%)	4 (9.80%)	0.347^a^

a Compared with group A, *P* > 0.05.

b Compared with group A, *P* < 0.05.

* indicated significant difference.

**Table 4:** Perioperative hematologic data of non-transfused patients with different fusion extent (based on lumbar fusion) (mean ± SD).

Hematologic data	LIV ≥ L1		*P* value	LIV < L1		*P* value
	Drainage	Non-drainage		Drainage	Non-drainage	
	(*n* = 8)	(*n* = 9)		(*n* = 27)	(*n* = 42)	
Hemoglobin (g/dL)						
Preoperative	131.5 ± 10.4	128.7 ± 9.3	0.562^a^	130.36 ± 7.27	128.94 ± 8.40	0.504^a^
0 days postoperative	106.06 ± 9.40	108.11 ± 6.58	0.607^a^	103.50 ± 11.84	104.18 ± 12.32	0.836^a^
3 days postoperative	102.50 ± 14.37	100.89 ± 8.62	0.780^a^	92.51 ± 7.64	99.12 ± 9.81*	0.008^b^
7 days postoperative	109.62 ± 13.76	107.33 ± 6.78	0.664^a^	108.25 ± 6.28	107.85 ± 9.36	0.847^a^
Hematocrit (%)						
Preoperative	38.65 ± 2.34	38.48 ± 2.97	0.897^a^	38.50 ± 3.17	38.82 ± 2.21	0.670^a^
0 days postoperative	31.43 ± 2.58	32.38 ± 2.13	0.417^a^	30.48 ± 3.38	31.04 ± 3.50	0.547^a^
3 days postoperative	29.91 ± 3.74	28.74 ± 2.85	0.918^a^	27.52 ± 1.93	29.79 ± 3.35	<0.01^b^
7 days postoperative	32.60 ± 4.30	31.96 ± 1.39	0.676^a^	32.45 ± 2.26	32.73 ± 2.70	0.679^a^

a Compared with group A, *P* > 0.05.

b Compared with group A, *P* < 0.05.

* Indicated significant difference.

**Table 5:** Postoperative clinical data of the patients with different fusion extent (based on lumbar fusion) (mean ± SD).

Postoperative data	LIV ≥ L1		*P* value	LIV < L1		*P* value
	Drainage	Non-drainage		Drainage	Non-drainage	
	(*n* = 8)	(*n* = 9)		(*n* = 27)	(*n* = 42)	
Length of stay (days)	8.6 ± 2.0	9.8 ± 4.0	0.464^a^	10.9 ± 3.8	7.7 ± 2.1*	<0.01^b^
Duration of pyrexia (days)	0.9 ± 0.4	1.2 ± 0.7	0.243^a^	0.9 ± 1.1	1.2 ± 0.1	0.335^a^
Peak temperature within 72 h ( °C)	37.1 ± 0.3	37.3 ± 0.3	0.413^a^	37.6 ± 0.4	37.7 ± 0.3	0.226^a^
Allogenic blood transfusion cases, *n* (%)	1 (12.5%)	1 (11.1%)	1.000^a^	7 (25.9%)	4 (9.5%)*	0.021^b^
Blood transfusion volume (mL)	25.00 ± 70.71	22.22 ± 66.67	0.935^a^	45.16 ± 88.84	21.05 ± 62.20*	0.035^b^

a Compared with group A, *P* > 0.05.

b Compared with group A, *P* < 0.05.

* Indicated significant difference.

### 3.4 Lumbar mobility and subjective pain in follow-up

**Figure 3:**
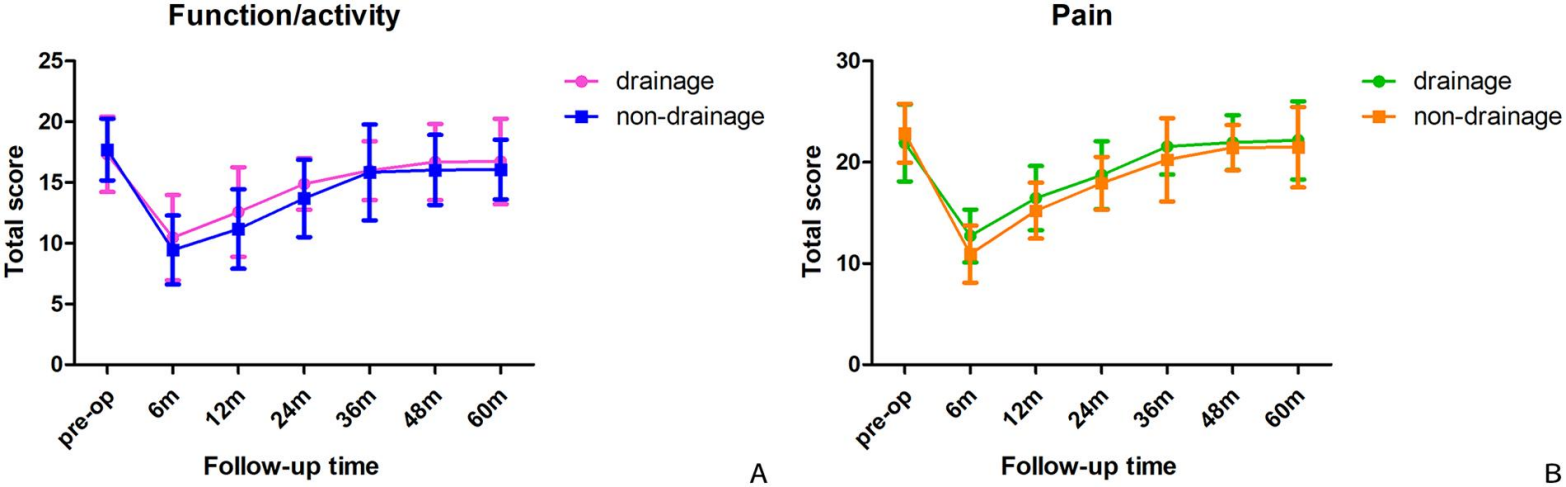
Total scores of the function/activity **(A)** and pain domains **(B)** were recorded at each follow-up. Although the non-drainage group showed slightly lower scores at the early stages, comparable scores were observed at the latest follow-up.

In general, the follow-up times of drainage and non-drainage group were 62.0 ± 18.0 months and 65.4 ± 11.6 months, respectively. No radiographic sign of calcification or ossification with high density was identified in paravertebral soft tissue on x-ray plain films at the latest follow-up, neither did we observe any internal fixation failure. No significant difference was found in lumbar mobility between the two groups (42.79 ± 13.81°vs. 44.97 ± 17.57°, *P* = 0.586) at the last follow-up. We further extracted and calculated the domain-specific scores on the SRS-22 questionnaires during regular follow-ups based on the patients' self-reported perceptions. Although trends of amelioration were witnessed, no significant differences were observed in the pain domain (21.92 ± 3.82 vs. 22.87 ± 1.92, *P* = 0.177) and the function/activity domain (17.32 ± 3.11 vs. 17.71 ± 2.54, *P* = 0.552) at the last follow-up (Table 6, Figure 3).

**Table 6:** Comparison of the SRS-22 scores of two groups at different times (mean ± SD).

SRS-22 questionnaire items	Preoperative		*P* value	Latest follow-up		*P* value
	Group A	Group B		Group A	Group B	
	(*n* = 35)	(*n* = 51)		(*n* = 35)	(*n* = 51)	
Function/activity	17.32 ± 3.11	17.71 ± 2.54	0.552^a^	16.74 ± 3.52	16.08 ± 2.46	0.285^a^
Item 5	4.35 ± 0.95	4.60 ± 0.82	0.209^a^	4.10 ± 1.05	3.81 ± 0.99	0.172^a^
Item 9	4.38 ± 0.84	4.21 ± 1.14	0.469^a^	4.10 ± 0.97	4.02 ± 0.88	0.670^a^
Item 12	4.32 ± 1.18	4.47 ± 1.01	0.553^a^	4.28 ± 1.05	4.22 ± 0.68	0.757^a^
Item 18	4.28 ± 1.01	4.42 ± 0.98	0.519^a^	4.26 ± 1.01	4.10 ± 0.82	0.395^a^
Pain	21.92 ± 3.82	22.87 ± 1.92	0.177^a^	22.18 ± 3.86	21.48 ± 3.98	0.374^a^
Item 1	4.38 ± 0.92	4.34 ± 0.71	0.861^a^	4.38 ± 0.78	4.18 ± 0.77	0.201^a^
Item 2	4.30 ± 1.11	4.50 ± 0.60	0.331^a^	4.48 ± 0.81	4.28 ± 0.78	0.214^a^
Item 8	3.92 ± 1.16	4.26 ± 0.76	0.135^a^	4.16 ± 0.84	3.92 ± 0.96	0.188^a^
Item 11	4.92 ± 0.35	4.94 ± 0.49	0.967^a^	4.76 ± 0.85	4.76 ± 0.96	0.924^a^
Item 17	4.40 ± 1.19	4.84 ± 0.68	0.081^a^	4.40 ± 1.34	4.34 ± 1.31	0.823^a^

a Compared with group A, *P* > 0.05.

With respect to the patients with lumbar segments unfused (LIV ≥ L1), no difference was observed in the latest lumbar mobility (49.62 ± 9.87° vs. 52.00 ± 15.60°, *P* = 0.717), function/activity domain (16.20 ± 2.63 vs. 15.71 ± 3.40, *P* = 0.227) and pain domain (20.58 ± 4.76 vs. 19.93 ± 3.21, *P* = 0.326), which is similar to the general comparison outcome. In contrast, for the patients with extensive lumbar fusion (LIV < L1), the drainage cohort presented a higher lumbar mobility than non-drainage cohort (45.59 ± 14.65° vs. 37.50 ± 13.60°, *P* = 0.032). In comparison, no difference was found specifically in function/activity or pain domain.

## 4 Discussion

### 4.1 Literature review

Until now, there has been no universal consensus regarding the usage of postoperative drainage in the AIS patients receiving posterior correction and instrumentation. Sathish Muthu performed a thorough systemic review of the relevant literatures till April 2020 and found no significant difference in postoperative infection rate and wound healing between drainage and non-drainage groups (16). Likewise, Liu JM revealed no significant association between wound drainage and wound infection, hematoma formation and postoperative blood transfusion volume in a meta-analysis including 1,295 patients from 5 retrospective studies (750 patients with drainage, 545 patients without drainage) (17). Liu Yancheng performed another systemic review and meta-analysis that included 1,756 patients from 8 clinical studies (1,072 patients with drainage and 684 without drainage) and reached a consistent conclusion with Liu JM (18). However, these aforementioned literatures failed to include well-planned prospective randomized comparative studies of high quality. This scarcity of well-planned RCT may be attributed to the various subspecialty training courses and the differing clinical instincts or preferences among surgeons. Spine surgeons tended to determine the drainage placement directly according to their rough judgment of the risk of wound complications. Moreover, the practical preference of their supervisors brought profound impacts on their choice of the postop drainage.

Apart from the meta-analyses with high evidence levels, retrospective clinical studies were also performed by different study groups. The conclusions of these studies were equally meaningful. Mohammad Diab carried out a multicenter retrospective study discussing the outcome of postoperative drainage on AIS patients receiving posterior surgeries. With a comparatively large sample size of 500 individuals, this study revealed no significant difference in the risk of surgical site infection and neurological complications in long-term follow-up (19). This conclusion was vigorously supported by Mohammad Sami who retrospectively investigated 402 patients receiving posterior laminectomy and interbody fusion (20). Meanwhile, both researches indicated that the patients with posterior drainage tended to need more blood transfusion therapy and nutrient supplementation (transfusion rate was 44.0% vs. 22.0% and 23.9% vs. 6.8%, *P* < 0.05) (20). These two researches reflected a widely accepted perspective that wound drainage tended to result in more postoperative blood loss. As a matter of fact, despite the application of tight suture and thick compressed dressings over the wound, residual cavities often inevitably persist within the surgical site. This phenomenon is particularly pronounced in regions with lumbar lordosis. The maintenance of a natural hematoma can facilitate early clot formation and exert a tamponade effect on potential bleeding sites, which might be disrupted by continuous suction drainage.

Given that the volume of the intraoperative blood loss within the surgical site was approximately equal, we were convinced that the Hb level could indirectly reflect the secondary blood loss after surgery. In our study, the non-drainage group showed a lower transfusion rate (9.8% vs. 20.0%) and higher 3-day Hb level (99.95 ± 9.45 g/L vs. 93.73 ± 10.08 g/L), which is in accordance with Diab's and Sami's studies. This phenomenon probably resulted from the extracorporeal compression on subtle bleeding points, which were responsible for the gradual formation of hematoma within the surgical site. We reasonably inferred that this compression was significantly weakened by submuscular drainage, which served as an effective outlet for the intracavitary hematoma.

### 4.2 Hematoma and neural complications

Some spine surgeons preferred to place wound drainage to prevent excessive compression on the nerve tissue from hematoma formation (21). This practice was especially common in nerve decompression surgery wherein extensive laminectomy and decompression maneuver were performed. However, the ultimate goal of mild to moderate AIS correction operation was to rebuild normal spine alignment and global balance, in which high-level osteotomy and extensive laminectomy and neural tissue exposure were always absent (22). In our study, few patients received extensive laminectomy, articular process resection and dural rupture. Thus, we speculated an extremely low incidence of hematoma formation.

An effective evaluation of hematoma formation is essential. Even though soft tissue MR and ultrasound examinations were not routinely performed after surgery, we can still estimate the probability of hematoma formation based on the presence of swelling, tenderness, ecchymosis, exudate and the healing condition of the surgical sites. In our study, the soft tissue condition was regularly assessed and recorded by two independent surgeons, which ensured the reliability of the outcomes. The results showed that neither incision-related complication nor radiographic calcification was observed during the follow-up. Thus, we confirmed that the incidences of hematoma were similarly low in the two groups and no clinically significant hematoma formed within the surgical site, which might resolve via absorption or organization.

After excluding the confounding factors, a conclusion could be safely reached that the postoperative drainage was not a determining factor for neural complications. As a matter of fact, spinal cord and nerve roots were more vulnerable to direct impacts such as clamping, cutting and dragging maneuvers rather than the gradual hematoma compression after surgery.

### 4.3 Lumbar mobility

Lumbar mobility is another important indicator for assessing the prognosis of posterior correction surgery. Several factors influencing lumbar mobility have been identified. Apparently, the number of preserved lumbar segments during surgery positively correlates with postoperative lumbar mobility (23, 24). Meanwhile, painful muscular spasm and paravertebral muscle fatigue are also predominant factors for postoperative mobility loss. From our perspective, muscular spasm could be majorly attributed to unintended muscle bundle damage during the exposure process and persistent instrument-related extraction throughout the whole surgery. Furthermore, secondary denervation-induced muscle atrophy was considered to be responsible for the irreversible paravertebral muscle fatigue. In addition, during the postoperative rehabilitation stage, patients preferred simpler physical exercise deliberately to avoid excessive lumbar motion, which somehow helped relieve the consistent muscle pain (25). All the aforementioned factors contributed to the long-term loss of lumbar mobility. In our study, all surgeries were performed by a single group of experienced spine surgeons with uniform techniques. Meanwhile, there was no significant difference in preoperative Cobb angles, curvature types and conserved segments between the two groups. Thus, intraoperative influences due to various exposure skills and surgical plans were minimized to the greatest extent.

### 4.4 Myositis ossificans

The development of paravertebral myositis ossificans was described as another important restricting factor for lumbar mobility (26, 27). Myositis ossificans is a non-neoplastic benign bone and cartilage pseudotumor that develops within skeletal muscles adjacent to the facet joints in lumbar regions. Although not clearly investigated, myositis ossificans has been reported to be closely related to local hematoma formation in correction surgeries (28). With the aggregation of inflammatory factors (such as IL-1 and PEG-2) through chemotactic response, the hematoma is gradually replaced by granuloma tissue and finally develops into a solitary lesion (29). Severe myositis ossificans is responsible for facet joints rigidity and further loss of spinal mobility. Therefore, we could safely establish a close relation between the surgical site hematoma and long-term lumbar mobility loss. In our study, all patients were closely observed for at least 5 years, far exceeding the time interval needed for myositis ossificans formation and maturity. Moreover, patients were required to follow standardized professional physical exercise instructions to improve lumbar mobility during rehabilitation. Hence, the different follow-up time intervals and individual exercise preference had no significant influence on lumbar function.

As LIV level has been identified by Ho C as a risk factor for delayed postoperative infection (16% with a thoracic LIV vs. 33% with a lumbar LIV) (30), we inferred that the extent of lumbar fusion would probably interfere with the outcome of wound drainage. Based on this assumption, we performed a subgroup analysis according to whether the lumbar segments were extensively fused or not. Subgroup A consisted of individuals with thoracic curve fused only. In contrast, subgroup B consisted of individuals with lumbar curve fused. In subgroup A, there were no significant differences in lumbar mobility, pain index domain and physical/social functioning domain of the SRS-22 questionnaire. However, in subgroup B, the long-term lumbar mobility of the drainage group was significantly greater than that of the non-drainage group. No difference was found in the pain index domain of SRS-22 questionnaire. The possible reasons are as follows: (1) the simpler anatomical structure and physical thoracic kyphosis made it easier to suture the surgical incision in the thoracic region with less subcutaneous cavity left. (2) the thick compressed dressings in thoracic region generated a more dominant hemostasis effect with the help of thoracic kyphosis and minimized the myositis ossificans formation. (3) the innate lumbar lordosis and larger laminar decortication area in lumbar segments made it comparatively harder to diminish the hematoma formation without wound drainage.

### 4.5 Limitations

This study has several limitations. Firstly, the study is not a double-blind randomized control trial (RCT). The patients were enrolled consecutively and were not randomly assigned to different groups, so the study efficacy is not comparable with RCTs. Secondly, the 7-day hemoglobin and hematocrit levels did not differ significantly between the two groups, which was inconsistent with the 3-day lab test results. We suspected that practicing physicians deliberately increased the postoperative fluid supplement volume for the patients with excessive drainage to prevent potential hypovolemic shock. Thirdly, a larger sample size is valuable to improve the efficacy and evidence level of this retrospective study.

## 5 Conclusions

This clinical study revealed the thick compressed dressing clothes without drainage was a clinically superior alternative to continuous wound drainage when considering postoperative blood loss and hospital stay. Despite these differences, neither long-term lumbar function nor chronic back pain was significantly affected by wound drainage. In the posterior fusion surgeries without extensive osteotomy in AIS patients, simple compressed dressing clothes without continuous submuscular drainage offered a clinically resource-efficient alternative, particularly in uncomplicated primary surgeries for mild to moderate deformity.

## Data Availability

The original contributions presented in the study are included in the article/Supplementary Material, further inquiries can be directed to the corresponding author.
